# Exchange-correlation functional's impact on structural, electronic, and optical properties of (N_2_H_5_)PbI_3_ perovskite

**DOI:** 10.1016/j.heliyon.2023.e17779

**Published:** 2023-06-29

**Authors:** Mohammad Tanvir Ahmed, Shariful Islam, Farid Ahmed

**Affiliations:** a Department of Physics, Jashore University of Science and Technology, Jashore, Bangladesh; b Department of Physics, Jahangirnagar University, Dhaka, Bangladesh

**Keywords:** DFT, Perovskite, Band gap, Exchange-correlation, Hydrazinium

## Abstract

One of the most popular multifunctional materials in optoelectronic research domains is organometallic perovskites. In this research, DFT calculation on Hydrazinium Lead Iodide (N_2_H_5_PbI_3,_ HAPI) perovskite with orthorhombic phase has been studied with distinct exchange-correlation functionals. HAPI showed a slight structural deformation using the LDA CAPZ functionals, revealing the minimum total energy. A very slight change in Mulliken and Hirshfeld charges of each element was observed due to the variation of functionals. The GGA calculations resulted in a perfect orthorhombic phase of HAPI, whereas LDA functional showed slight deformation from the orthorhombic phase. The band gaps of 1.644, 1.633, 1.618, and 1.650 eV were obtained using GGA (PBE, PBEsol, PW91) and LDA (CAPZ) functionals, respectively. HAPI showed a high absorption coefficient of 10^4^ cm^−1^ order with strong absorption of high energy visible wavelength. A maximum refractive index of 2.8 was observed in the visible wavelength region and a high optical conductivity of over 10^15^ s^−1^ suggests that HAPI can be a potential material for numerous optoelectronic research.

## 1 Introduction

A typical organometallic perovskite (OMP) has the general formula of ABX_3_, where A, B, and X stand for organic cation (monovalent), metal cation (divalent), and halogen anion, respectively [1]. These compounds are multifunctional with a wide range of applications, including in optical detectors, field-effect transistors, solar cells (SCs), LASER, light-emitting diodes, electrochemical cells, sensors, and more [2]. The perovskite solar cells achieved up to a power conversion efficiency (PCE) of 26.1% within a few years through remarkable improvement [3].

The biggest problem with OMPs is that they aren't durable in environments which include oxygen, humidity, electric fields, warm temperatures, light, and substances that resemble light. This problem could be partially resolved by compositional engineering. Only certain configurations of the A, B, and X ions can produce the complex ABX_3_ halide perovskite structure. The tolerance factor (τ) is one criterion proposed by Goldschmidt in the early 1920s to understand and quantify which mixtures of compounds may produce a stable perovskite crystal structure [4]. The tolerance factor of a perovskite structure can be obtained from equation (1).

(1) $$\tau =\frac{R_{A}+R_{x}}{\sqrt{2}\;\left(R_{B}+R_{x}\right)}\text{,}$$

where R_A_, R_B,_ and R_X_ represent the ionic radii of A, B, and X-site atoms, respectively. In contrast, if "τ" does not fall within the range of 0.81–1.11, a lower-dimensional connection of BX6 would result instead of a three-dimensional structure [4].

The OMP with the highest degree of research is CH_3_NH_3_PbI_3_ (MAPI) with τ = 0.91 and contains the methylammonium (CH_3_NH_3_^+^, MA^+^) of ionic radius 2.17 Å as an A-site cation [5]. Due to the simple and economical fabrication process, MAPI is the most commonly used OMP in various optoelectronic research [[6], [7], [8]]. The OMPs CH_3_NH_3_PbX_3_ (X = I^−^, Br^−^) were first synthesized for solar cell application by A. Kojima et al. in 2009, which showed up to 3.8% PCE [9]. Formamidinium lead iodide (FAPI) perovskites have a tolerance factor ⁓1, most commonly used for solar cell applications [[10], [11], [12]]. It was first synthesized by T. M. Koh et al. in 2013, showing a comparatively lesser band gap (1.43–1.48 eV) than MAPI [13]. FAPI also has an enhanced light-absorbing capability and achieved up to 25.7% PCE in SCs technology [14,15].

Another OMP [Et_3_NCH_2_Cl)PbBr_3_] with new A-site material was synthesized by Z. Cai et al., which demonstrated a higher band gap of 3.57 eV with a strong fluorescence under UV irradiation [16]. Ethylammonium (EA^+^) is also a potential candidate for the A-site cation with an ionic radius of 2.3 Å, comparatively higher than MA^+^ [17]. EAPbI_3_ perovskite-based SC was first synthesized in 2012, which showed a very poor PCE of 2.4% [18]. Organic perovskites with mixed MA^+^ and formamidinium (FA^+^) A-site cations have also been studied, showing better environmental stability, enhanced characteristics, and improved device performance [15,[19], [20], [21], [22]]. Rebecca et al. synthesized *n*-butylammonium incorporated MAPI, which increased the optical band gap with a blue shifting in photoluminescence intensity [23].

Meanwhile, the hydrazinium ion (N_2_H_5_^+^, HA^+^) is a potential candidate for the A site ion, which has a different geometry from MA^+^ but a similar ionic radius [24]. The hydrazinium lead iodide (N_2_H_5_PbI_3_, HAPI) has a tolerance factor 0.912 and hence can show fine structural stability. There have been incredibly few studies on HAPI reported up to this point. In optoelectronic (OE) research, HAPI is a stable perovskite structure and a substitute for MAPI, as predicted by theory [25]. In 2016, Akbulatov et al. fabricated the MA_1-x_HA_x_PbI_3_ perovskites with a hexagonal symmetry of HAPI for SCs active layers where the MA_0.9_HA_0.1_PbI_3_ combination showed the highest PCE [24]. The hexagonal HAPI was explored theoretically and experimentally by Campbell et al. (2018), where they acquired band gaps from 2.48 eV to 2.70 eV, which is relatively larger for SCs light-harvesting material [26]. The cubic symmetry of HAPI was also suggested through the research. According to Tsarev et al. partial replacement of MA^+^ by HA^+^ in Sn-based OMP can enhance device performance by reducing voids in the morphology [27]. Lin et al. (2019) synthesized Sn-based OMP from HASnI_3_ through cation displacement, which improved device performance with reduced Sn^4+^ [28]. However, other phases of HAPI (e.g., cubic, tetragonal, orthorhombic) have not been studied previously.

Here, we designed and investigated orthorhombic HAPI perovskite's geometrical, electronic, and optical properties via density functional theory (DFT) calculations. We implemented four distinct exchange-correlation functionals throughout the DFT calculations and studied the variation in different properties of HAPI. To our knowledge, a similar study on orthorhombic HAPI was not reported earlier. This investigation revealed that HAPI perovskite is a potential substitute for MAPI in various OE research.

## 2 Computational details

The DFT calculation has been carried out through the “CAmbridge Serial Total Energy Package (CASTEP)" using ultrasoft pseudopotential. The contribution of Iodine (I) and Lead (Pb) atoms' valence electrons are taken into consideration for calculating OE properties. For geometrical optimisation, the Broyden–Fletcher–Goldfarb–Shanno (BFGS) algorithm is used [29]. We have utilised the local density approximation (LDA) functional CAPZ (Ceperley–Alder–Perdew–Zunger), generalised gradient approximation (GGA) functionals PBE (Perdew-Burke-Ernzerhof), PBEsol (revised PBE functional for solids), and PW91 (Perdew-Wang) for exchange-correlation corrections [30]. We designed a 2 × 2 × 1 supercell of HAPI structure where a 3 × 3 × 3 k-point mesh following the scheme of Monkhorst-Pack and a plane wave cut-off energy of 500 eV was used throughout the simulation process [31]. Geometry optimisation was achieved successfully using convergence criteria of pressure to be 0.05 GPa, 3 × 10^−2^ eV/atom for maximum force, and 0.001 Å for displacement [31].

## 3 Results and discussion

### 3.1 Geometrical analysis

**Fig. 1:**
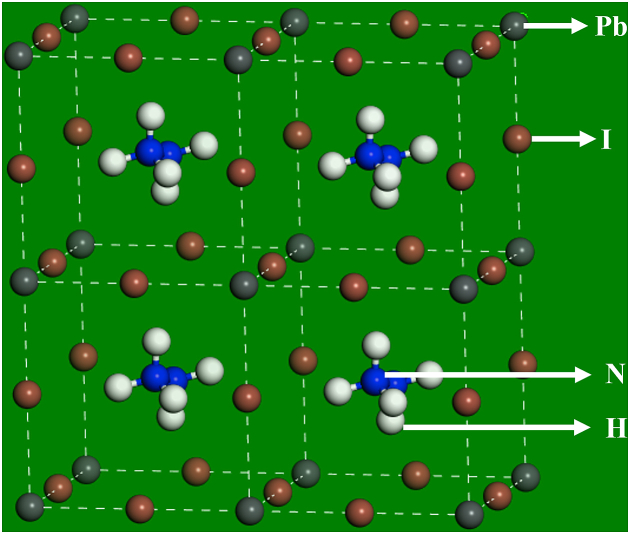
Geometrical structure of HAPI perovskite.

The HAPI perovskite is designed as similar to MAPI (replacing CH_3_NH_3_^+^ by N_2_H_5_^+^ ion) of space group Pm3 m [32]. The geometrical structure of the optimised HAPI structure is shown in Fig. 1. The variation in lattice parameters and the total energy calculated via different functionals is listed in Table 1. It is observed that the minimum total energy is obtained by employing the LDA CAPZ functional; however, the structure showed slight deviation from the orthorhombic to triclinic crystal phase. The PBE, PBEsol, and PW91 functional reveal an orthorhombic phase of HAPI perovskite. Since LDA shows higher structural deviation from the experimental values compared to GGA [33], the optimised structures using GGA are thought to be more precise. The LDA results in smaller lattice constants compared to that of GGA, which satisfies previous studies [34,35]. HAPI can show almost similar structural stability to MAPI, with a tolerance factor of 0.912 [24].

**Table 1:** Unit cell parameters and minimum energy for different functionals.

Functional	a, b, c (Å)	α, β, γ (Degree)	Volume (Å^3^)	Ground state energy (eV)
LDA CAPZ	6.215, 6.162, 6.159	86.39, 88.59, 91.95	235.226	−3231.845
GGA PBE	6.469, 6.419, 6.375	90, 90, 90	264.709	−3224.205
GGA PBEsol	6.335, 6.291, 6.265	90, 90, 90	249.585	−3216.739
GGA PW91	6.458, 6.409, 6.370	90, 90, 90	263.676	−3226.888

### 3.2 Electronic properties

#### 3.2.1 Mulliken charges analysis

The charge distribution of the HAPI structure is obtained using the Mulliken and Hirshfeld charge analysis [36,37]. The average Mulliken charge of the elements in the HAPI perovskite obtained using different functionals is listed in Table 2. Since N and I atoms have strong electronegativity, they exhibit a partial negative charge in the configuration. Pb and H atoms, in contrast, have a partial positive charge, which means that bonding electrons are moved from Pb and H atoms and are now bound to I and N atoms, respectively. The Mulliken charges show very slight variation for different functionals. The average Hirshfeld charge follows the analogous pattern (Table 3), i.e. both N and I atoms show partial negative charges, whereas H and Pb atoms show partial positive charges.

**Table 2:** Average Mulliken charges obtained from different functionals.

Elements	LDA CAPZ	GGA PBE	GGA PBEsol	GGA PW91
H	0.378	0.325	0.38	0.38
N	−0.66	−0.62	−0.66	−0.66
I	−0.42	−0.343	−0.44	−0.437
Pb	0.69	0.73	0.74	0.73

**Table 3:** Average Hirshfeld charges obtained from different functionals.

Elements	LDA CAPZ	GGA PBE	GGA PBEsol	GGA PW91
H	0.098	0.102	0.102	0.102
N	−0.07	−0.065	−0.065	−0.065
I	−0.207	−0.213	−0.22	−0.213
Pb	0.27	0.26	0.26	0.26

#### 3.2.2 Band gap analysis

**Fig. 2:**
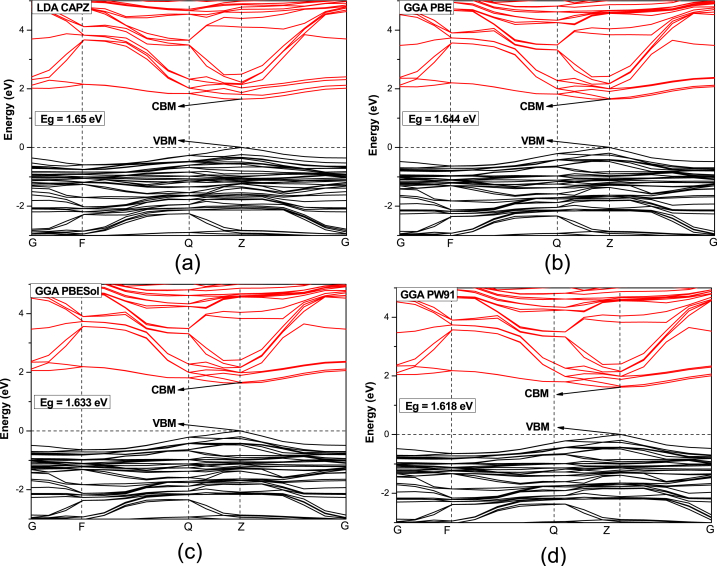
Band structure of HAPI obtained using (a) LDA CAPZ, (b) GGA PBE, (c) GGA PBEsol, and (d) GGA PW91 functionals.

The band structure of the HAPI perovskites is shown in Fig. 2. Along the symmetry points of the Brillouin zone (G →F → Q → Z → G), the band structures have been determined. All the functionals computed a direct band gap of HAPI with both valence band maximum (VBM) and conduction band minimum (CBM) at the “Z" k-point, which proves that HAPI is a direct band gap (Eg) semiconductor. Using different functionals, the band gap was obtained from 1.618 eV to 1.65 eV. The band gap is close to the ones of MAPI and in the lower energy region of the visible spectrum, making HAPI a potential material for various optoelectronic applications [4], whereas significantly lower than the hexagonal phase of HAPI [26]. However, both LDA and GGA underestimate band structures, which can be the reason for the lower obtained band gap compared to the experimental results [38,39]. Compared to FAPI, the obtained bandgap of HAPI is significantly higher [40]. The lowest value of the band gap (1.618 eV) was obtained using GGA PW91 functional, whereas LDA CAPZ revealed the maximum band gap (1.65 eV). Since the material processes direct band gap, it can absorb electromagnetic waves more effectively compared to indirect band gap semiconductors.

#### 3.2.3 Density of states (DOS)

**Fig. 3:**
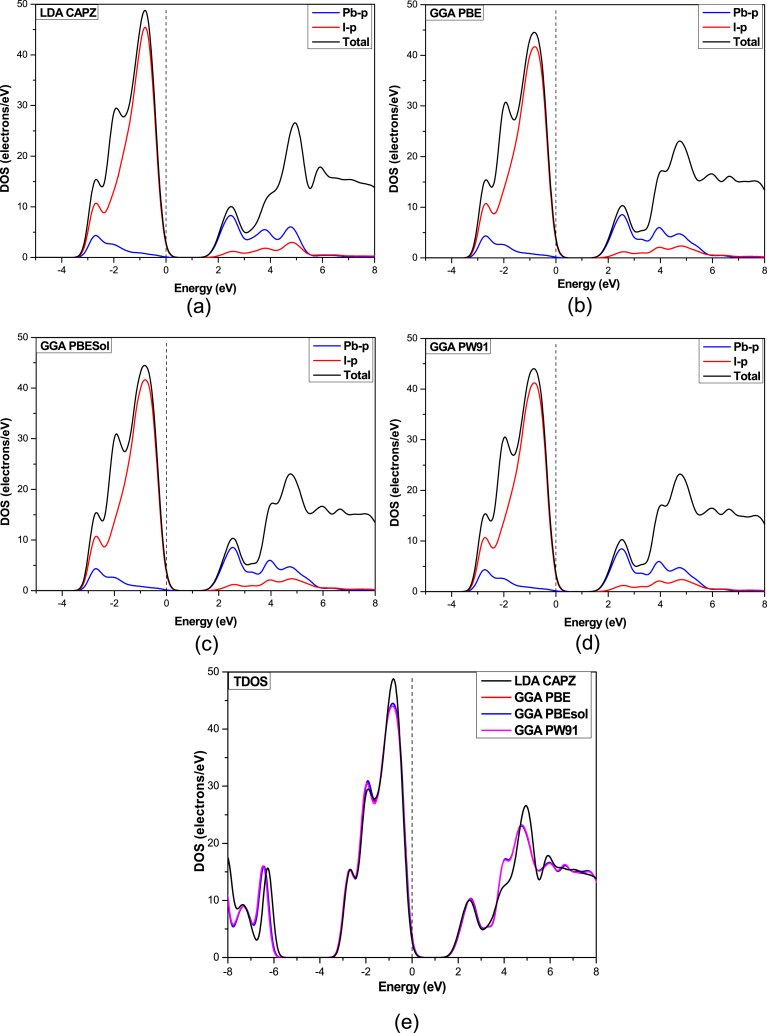
Partial density of states of HAPI using (a) LDA CAPZ, (b) GGA PBE, (c) GGA PBEsol, (d) GGA PW91 functionals, and (e) total density of states of HAPI.

The partial density of states (PDOS) and Total density of states (DOS) of HAPI are shown in Fig. 3. In HAPI perovskite, the electron configuration of Lead (II) is 6s^2^6p^0^ and 5s^2^5p^6^ for Iodine. Hence the p-orbital of Iodine significantly contributes to the VBM. According to previous studies, the crystal band edge is not directly affected by A-site cations; instead, they influence the change in crystal size and band gap [41]. Fig. 3 shows that the 5p orbital of Iodine atoms shows the maximum contribution to the valence band (VB), whereas the conduction band (CB) is composed of the 6p orbital of Lead atoms. This contribution to the CBM and VBM is almost analogous to all other perovskites [41]. No significant changes in the contribution to VB and CB are observed due to the variation of functionals which was also observed in a previous study [42]. The observed TDOS values in LDA are slightly higher compared to those in GGA, which satisfies the previous report [42].

### 3.3 Optical properties

**Fig. 4:**
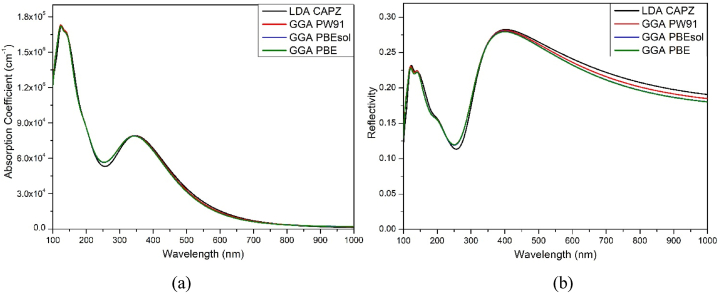
(a) Absorption coefficient and (b) reflectivity of HAPI perovskite.

Very slight changes in the optical properties of HAPI are observed due to the variation of functionals. The optical reflectivity and absorption coefficient (AC) of the modelled perovskite is shown in Fig. 4. HAPI showed an extremely strong AC (over 10^4^ cm^−1^) in the shorter wavelength of the visible region, making them perfect for various OE applications [[43], [44], [45], [46]], e.g., LASER, solar cells, LED, etc. However, the AC is comparatively lower than the MAPI perovskites observed in theoretical and practical studies [[47], [48], [49]]. The absorption curve is analogous to previous research [24]. A high absorption coefficient represents the lower penetration depth (δ) of electromagnetic waves in the sample [50]. For example, the values of AC near 400 nm and 750 nm are about $7\times 10^{4}\;cm^{-1}\;and\;5\times 10^{3}\;cm^{-1}$, corresponds to the penetration depths of 0.14μmand2μm, respectively. The incident 400 nm wavelength absorbed by 37% after traversing only 0.14μm thick HAPI sample, which means only a few micron thickness of HAPI is enough for absorbing most of the visible spectrum. No significant effect of the variation of functionals are observed in the AC of HAPI.

A crucial characteristic for OE applications is reflectivity, which measures the percentage of energy reflected from crystal surfaces. Due to decreased energy loss, a material with lower reflectivity can perform better in OE applications [51]. HAPI shows a maximum reflection of blue light, maintaining an overall reflection below 30% in the visible wavelength region. The reflectivity slightly decreased with the increase of wavelength in the visible region. Slightly higher reflectivity was observed in the visible wavelength region via LDA. A very minor variation/shifting in the AC or reflectivity is observed with the variation of functionals.

**Fig. 5:**
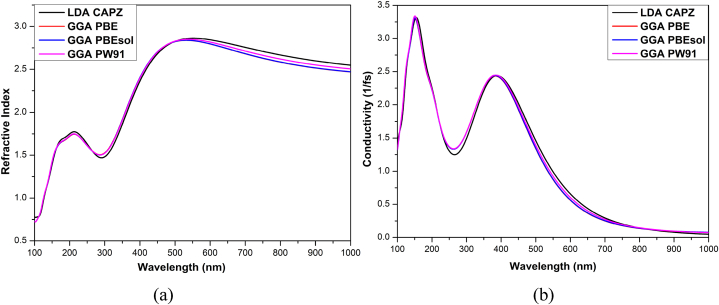
(a) Refractive index and (b) optical conductivity of HAPI.

The HAPI perovskite shows a maximum of 2.8 refractive indexes (*η*) at 545 nm wavelength (Fig. 5). The values of *η* ranged from 2.43 to 2.8 in the visible region, slightly higher than MAPI [52] signifies that more reflection of the incident wave will occur from the surface of HAPI compared to MAPI. The refractive index showed an almost similar effect of functional variation as observed in the reflectivity. The reduction of *η* in the visible range via different modifications of the HAPI structure may result in better optoelectronic performance [53].

The increase in electrical conductivity brought on by photon absorption is represented by optical conductivity (OC). Hence, OC is a representation of photoconductivity [54]. Due to increased energy, the highest conductivity is seen in the blue wavelength area. In the visible region, the maximum OC is about 2.45 fs^−1^ at 390 nm wavelength. OC spectra in the visible region are quite similar to MAPI; however, the value of OC is slightly lower [55]. The optical conductivity of HAPI is significantly high for various OE purposes. The OC decreased with the decrease in incident photon energy, as observed for MAPI [55].

**Fig. 6:**
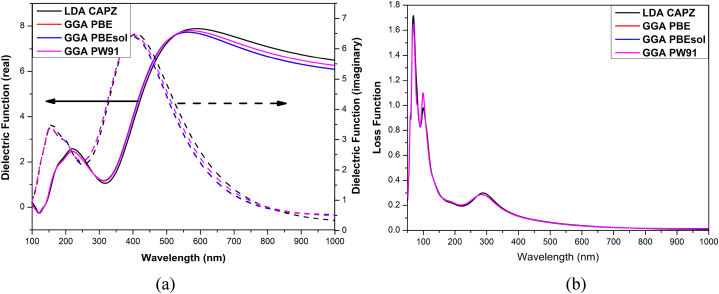
(a) Dielectric function and (b) loss function of HAPI.

Fig. 6 shows the dielectric function (real and imaginary) and loss function of the HAPI perovskite. The imaginary portion indicates energy dissipation, whereas the real part represents polarisation inside the material [56]. The imaginary portion of the dielectric function directly determines the absorption property of HAPI; hence analogous to the absorption spectra. The imaginary part of the dielectric function shows the maximum value at 403 nm, which further decreases with increasing wavelength. In contrast, the real part of the dielectric function is dominant in the overall visible region. The maximum real dielectric constant is observed in the visible region (at 550 nm), which further decreased with decreasing photon energy as observed MAPI structure [55].

The energy loss function (LF) represents the plasmonic oscillations in the structure. Light contact with matter creates plasma oscillations when electrons are not bound to a lattice site. These oscillations are accountable for loss function peaks known as plasmon peaks [57]. The plasmon peak of the HAPI structure is observed in the UV and X-ray regions. The major peak of LF is observed at 69 nm wavelength. The LF peak near 290 nm is analogous to the MAPI structure [58]. No significant shifting of the plasmon peak is observed due to the variation of the functionals.

## 4 Conclusion

The geometrical, electronic, and optical properties of orthorhombic HAPI perovskites and their variation with different functional employment have been studied via DFT analysis. The HAPI perovskite can show better structural stability than MAPI due to the suitable tolerance factor. The GGA PBE, GGA PBEsol, and GGA PW91 provided the perfect orthorhombic phase of HAPI, whereas LDA CAPZ functional developed the maximum deformed geometry from the orthorhombic phase with the minimum ground state energy. A minimal variation in surface charge distribution with a slight change in band gap was observed while analyzed using distinct functionals. The band gap is observed in the range of 1.618–1.650 eV due to the change in functionals, which is suitable for SCs application. The optical properties showed minimal variation while changing the functionals. The high absorption coefficient over 10^4^ cm^−1^ makes HAPI a potential light-harvesting material for SCs. Both reflectivity and refractive index showed significant energy loss of photons, which can be further improved via modification of the HAPI structure. The HAPI structure is highly photoconductive, which makes HAPI suitable for numerous OE applications.

## Author contribution statement

Mohammad Tanvir Ahmed: Conceived and designed the experiments; Performed the experiments; Wrote the paper.

Shariful Islam: Analyzed and interpreted the data.

Farid Ahmed: Contributed reagents, materials, analysis tools or data.

## Data availability statement

Research data is not shared because they are currently in use in another study.

## Declaration of competing interest

The authors declare that they have no known competing financial interests or personal relationships that could have appeared to influence the work reported in this paper.
